# The Role of Tumor-Derived Exosomes in the Abscopal Effect and Immunotherapy

**DOI:** 10.3390/life11050381

**Published:** 2021-04-23

**Authors:** Zechen Shan, Hongmei Wang, Yujuan Zhang, Weiping Min

**Affiliations:** 1 Academy of Queen Mary, Nanchang University, Nanchang 330000, China; 6303416152@email.ncu.edu.cn (Z.S.); yujuanzhang@ncu.edu.cn (Y.Z.); 2 School of Basic Medical Sciences, Nanchang University, Nanchang 330000, China; 3 Department of Surgery, Pathology and Oncology, University of Western Ontario, London, ON N6A 5A5, Canada

**Keywords:** tumor-derived exosomes, biomarkers, immunomodulation, abscopal effect, radiotherapy

## Abstract

Exosomes are microvesicles that can be secreted by various cells and carry a variety of contents; thus, they play multiple biological functions. For instance, the tumor-derived exosomes (TEXs) have been proven to have the effect of immunostimulatory in addition to immunosuppression, making TEXs attractive in clinical immunotherapy and targeted therapy for cancer patients. In addition, TEXs as biomarkers have important clinical diagnostic and prognostic value. Recently, TEXs have been recognized to play important roles in the abscopal effect (AbE), a newly discovered mechanism by which the distant tumors are effectively targeted and repressed during immunotherapy and radiotherapy. Therefore, TEXs has demonstrated great clinical potential in the diagnosis, prognosis and treatment of cancer patients in the future. This review summarizes and discusses the role of TEXs in clinical therapy and their role in AbE in recent studies.

## 1. Introduction

The abscopal effect (AbE) refers to the regression of a distant tumor away from the primary tumor due to the therapeutic effect of immunotherapy or radiotherapy. The concept of the AbE was first reported by Mole RH in 1953, who described the AbE as the regression of other tumor foci that were outside the initial localized radiation treatment field [1]. Over the past decades, the mechanism of the AbE has been elucidated and it was proved that this process was mediated by the immune system leading to tumor cell death, which involves T regulatory cells, suppressor cells, dendritic cells and the ligands/receptor of dendritic cells as critical mediators [2]. Upon the accumulation of AbE in different cases, more evidence showed that the immune system plays an important role in regulating the AbE.

Radiotherapy is one of the most common cancer therapies nowadays, and it is used in the curative and palliative treatment of various cancer for local-regional control [3]. More recently, radiotherapy has been shown to promote some systemic immune modulatory effects including the AbE on the tumor patients, by which a local radiation treatment was found to elicit a systemic immune response and to release the malignant burden to untreated tumor areas [4]. Though the AbE was proposed decades ago, it still remains to be fully understanding for its roles and mechanism in the immunomodulation and immunotherapy of cancer, whereas accumulating evidence has revealed that the AbE is greatly associated with tumor exosomes.

Exosomes are subgroup of extracellular vesicles, which are released by all types of cells including tumor cells. The molecules carried by exosomes play an important role in intercellular communication. Studies have been focused on the biological and physiological functions of exosomes: immune activation, cancer therapies and being biomarkers. The exosomes have immunological activities including modulating antigen presentation, immune activation, immune suppression, immune surveillance and intercellular communication, which all affect immune-regulating mechanisms. Emerging evidence has shown that tumor-derived exosomes (TEXs) carry both immunostimulatory and immunosuppressing functions, which depend on what molecules are inside the exosomes and the state of the immune cells in the tumor microenvironment [5]. Studies have also demonstrated that exosomes have immunotherapeutic applications. This indirectly proved that TEXs carry both immunostimulatory and immunosuppressive ligands/receptors that partly mimic the profiles of the parent tumor cells [6].

Intercellular communication plays an important role in proliferation and metastasis of cancer cells, and this process could be triggered by growth factors, cytokines, tumor-associated antigens and some other molecules [7]. TEXs can serve as regulatory agents responsible for the interaction between cancer cells and the microenvironment. Evidence revealed that exosomes released from tumor cells may not only affect proximal tumor cells and stromal cells in the local microenvironment, but can also set off systemic effects as they circulate in the blood carrying the functional components like microRNAs (miRNAs) and proteins. This type of TEXs promotes the tumor growth, invasion and metastasis of cancer, which are all related to the AbE mentioned above. It has now been widely recognized, in a number of recent studies, that TEXs are capable of boosting tumor growth as well as promoting the progression and metastasis of cancer through suppressing or modifying the immune response towards cancer cells, regulation of tumor neo-angiogenesis and pre-metastatic niche formation [8].

Recent studies have also demonstrated that TEXs separated from tumor cells could serve as tumor biomarkers because of the TEXs transferring and containing various tumor-related materials, including nucleic acids, glycoconjugates and lipids, which partially reflect the molecular and genetic content of the parent cancer cell. According to this, exosomes are being recognized as very helpful biomarkers for the prognosis, diagnosis and therapeutic response of cancer [8].

## 2. Tumor-Derived Exosomes (TEXs)

### 2.1. Exosomes

Exosomes are a kind of extracellular vesicles that are formed inside the cells and secreted from the cells through exocytosis. Exosomes differ from microvesicles larger than 100 nm in diameter, with an average diameter of 30–100 nm. The biogenesis of exosomes starts from the invagination of the plasma membrane to form endosomes controlled by the endosomal system. Then, during the process with the participation of Golgi bodies, exosomes are formed from endosomes in the early phase to multivesicular bodies in the late phase, and the membrane of multivesicular bodies could form a lumen where intraluminal vesicles are contained. Then the multivesicular bodies could be degraded by lysosomes or fused with plasma membrane, and the intraluminal vesicles could be released outside the cell through exocytosis to form exosomes.

The process above is regulated by Rab GTPases and the endosomal-sorting complex required for transport (ESCRT) system. The Rab GTPases in this study included Rab14, Rab22, Rab27 and Rab37, which were responsible for the formation of intraluminal vesicles, the fusion of the multivesicular bodies with the plasma membrane and the degradation of the multivesicular bodies by lysosomes. The ESCRT included ESCRT-0, ESCRT-I, ESCRT-II and ESCRT-III. ESCRT-0 was responsible for assembling molecules and internalizing the ubiquitinated proteins. ESCRT-1 and ESCRT-II were responsible for the invagination process and promoting the deubiquitination of those ubiquitinated proteins. ESCRT-III was responsible for the separation and disassembling of intraluminal vesicles [9].

**Figure 1:**
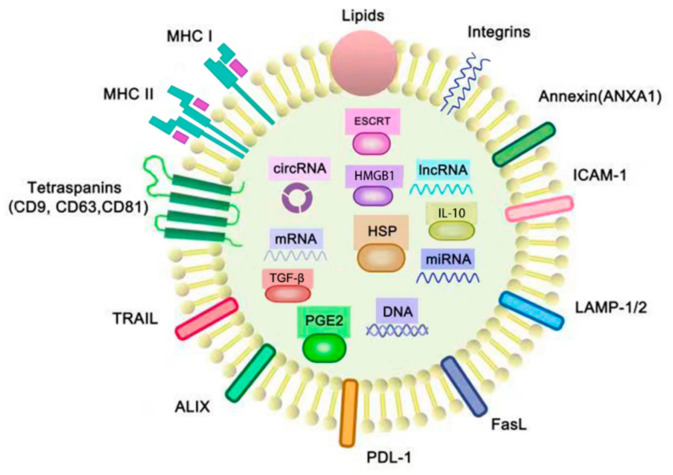
Structure and cargoes of exosomes. The membranes of exosomes consist of phospholipid bilayers, proteins and lipids, and there are many molecules contained in the aqueous core of exosomes. These molecules include the cytoplasmic proteins: the endosomal-sorting complex required for transport (ESCRT), prostaglandin E2 (PGE2), heat shock protein (HSP), high-mobility group box 1 (HMGB1), TGF-β and IL-10; the membrane proteins: integrins, intercellular adhesion molecule 1 (ICAM-1), lysosomal-associated membrane glycoprotein 1/2 (LAMP-1/2), FasL, PDL-1, ALIX, TRAIL, tetraspanins (CD9, CD63 and CD81), major histocompatibility complexes I and II (MHC I, II) andANXA1; and the nucleic acids: miRNA, mRNA, circRNA and lncRNA. FasL, PD-L1, ALIX, TRAIL, PGE2, IL-10, TGF-β and tumor-associated antigens (TAAs) are typically found in TEXs for immunosuppression, whereas HSPs like HSP 70 and HSP 80, MHC molecules and tetraspanins are found in TEXs which are related with the functions of immunostimulatory. ANXA1 is a kind of annexin that not only plays a key role in cell–cell adhesion, but also inhibits tumor-induced inflammatory responses.

The exosomes only can be observed under the electron microscopy. Although there are many kinds of exosomes which are composed of different molecules that they transfer, the morphology of the exosomes is similar. Morphologically, the exosomes in our study were round in shape, with a phospholipid bilayer membrane and aqueous core containing proteins and nucleic acids. The molecules contained in exosomes were dependent on their parental cells, their pathophysiological conditions and environmental stimulations. Generally, as shown in Figure 1, the content of exosomes includes major histocompatibility complex class I and II (MHC I and II), heat shock proteins (HSP), tetraspanins (such as CD9, CD63 and CD81), integrins, intercellular adhesion molecule 1 (ICAM-1), lysosomal-associated membrane glycoprotein 1/2 (LAMP1/2), nucleic acids including miRNA, lncRNAs, mRNA and DNA, cytoplasmic proteins and other membrane-bounded proteins [9]. In addition to these proteins and nucleic acids, there were also cholesterol, sphingomyelin, phosphatidylethanolamine and phosphatidylcholine on the cell membranes of the exosomes in our study [10].

### 2.2. Tumor-Derived Exosomes (TEXs)

TEXs contain some extra immune suppression-associated molecules, such as tumor-associated antigens, FasL, PD-L1, ALIX, TRAIL, IL-10, TGF-β and prostaglandin E2 (PGE2) [5], which are immunosuppressive molecules and were shown to induce the inhibition of dendritic cells, the differentiation apoptosis of T cells, the differentiation of Tregs and the induction of myeloid-suppressive cells. On the other hand, TEXs are composed of immune stimulatory molecules such as co-stimulatory molecules and MHC molecules. Therefore, TEXs possess dual signaling abilities including immunostimulatory and immunosuppression [11].

The main functions of TEXs are determined by the role they play in the tumor microenvironment. Therefore, because of the abilities of intercellular communication and immune regulation, TEXs play a major role in cancer progression by the bioactive molecules interacting with the recipient cells and changing their functions. Research has demonstrated that TEXs are the carriers of oncogenetic signals and oncogenes to promote the process of neoplastic transformation. In general, TEXs were proven to be related to tumor progression, angiogenesis, drug resistance and immune regulation. Specifically, TEXs promote tumor progression through the upregulation of the tumor cell proliferation and tumor growth, the enhancement of tumor invasion and tumor metastasis, the suppression of anti-tumor immune responses and the promotion of the horizontal transfer of oncogenic mutations [12]. Epidermal growth factor (EGF-EGFR) and phosphatase and tensin homolog deleted on chromosome 10 (PI3K/AKT—PTEN) in TEXs are mostly involved in metastasis [13], and drug resistance including the removal of toxic drugs [14] and exchange of drug transporters [15]. Transportation of multidrug resistance (MDR)-associated miRNAs by TEXs have been associated with the elevation of drug resistance [13]. The angiogenesis is also involved in this process of TEX-based regulation through the following signaling pathways. The vascular endothelial growth factor (VEGF-VEGFR), TGF-β, and fibroblast growth factor (FGF-FGFR) in TEXs are mostly involved in angiogenesis [16].

### 2.3. Isolation of TEXs from Cancer Patients

Research has shown that most of the tumor-derived exosomes are isolated from the supernatants of cultured tumor cells [5]. Various methods used to isolate TEXs from the body fluid have been developed, which include complicated procedures of purification and recovery. For example, TEXs have been isolated by ultracentrifugation, filtration, immunoaffinity-based isolation and microfluidics techniques [17]. Ultracentrifugation is the most commonly used method to separate the exosomes, which based on the size and buoyant density. However, ultracentrifugation is time consuming, and has low recovery and low specificity. Ultracentrifugation also requires expensive equipment and specific technicians.

The density-gradient separation is another conventional method with a better purity and recovery rate for isolating exosomes. This separation method is based on the isopycnic point mechanism [10]. Although this method can achieve the higher recovery, purity and specificity, the disadvantages are almost the same as ultracentrifugation, such as being time consuming and requiring expensive equipment.

Immunoaffinity-based isolation is a method that depends on specific proteins on the membrane of exosomes. Antibody-conjugated beads are used in this method to recognize the specific antigens on the exosomes membrane and then capture the TEXs. This technique has more advantages: the reduction of cell-debris and protein aggregates in co-purification, better isolation of exosomes subgroups based on the specific antigens on the exosomes membranes, increased recovery rate and the ability to process multiple samples simultaneously [18,19].

There have also been other techniques used for isolation of exosomes, such as using the force of inertia in microfluidic channels to separate the exosomes from the other substances [10] and the lectin-induced aggregation of exosomes, which has a lower cost and easy operation [20]. Although these exosome isolation methods are practical for using in clinical practice, the problems in the isolation of TEXs still need to be properly resolved, including choosing the proper donor cells, the method of purification and amplification, cost and time [21]. Thus, techniques need to be further developed in order to isolate and handle TEXs properly and quickly in clinical settings.

## 3. TEX-Containing Genetic Materials That Are Associated with Cancer Progression and Prognosis

### 3.1. microRNA (miRNA)

miRNAs have been recognized as biomarkers in the diagnosis and prognosis of cancers. Studies in hepatocellular carcinoma showed that exosomal miRNA from serum-like miR-103 and miR-638 were potential biomarkers. Elevated miR-103 in hepatocellular carcinoma cells indicated metastasis, while decreased miR-638 was associated with late-stage cancer and the recurrence of cancer [22,23]. In colorectal cancer, studies found that miR-1229, miR-1246, miR-150, miR-21, miR-223 and miR-23a were mostly used for early diagnosis [24], whereas miR-17-92a and miR-19a were related to recurrence [25]. In lung cancer, the elevation of miR-217 and miR-4257 was related to the recurrence [26], and the decrease of miR-51 and miR-373 was related to a poor prognosis, and miR-208a and miR-1246 were the biomarkers of therapeutic effect [27]. In addition [28], miR-1246 and miR-4644 were elevated in pancreatobiliary tract cancer patients and associated with a poor prognosis [29].

### 3.2. Long Noncoding RNA (lncRNA)

lncRNAs were regarded as potential biomarkers in clinical applications, as was reported recently, and they also play a crucial role in the tumor microenvironment. In hepatocellular carcinoma, lnc-sox2ot [30], lnc-h19 [31] and lncRNA-ARSR [32] were shown in the recent studies to be associated with prognosis and survival. An elevated level of lncRNA-UCA1 in bladder cancer exosomes could be used for diagnosis and monitoring tumor progression [33]. Besides, it could be combined with PCAT-1 and MALAT-1 to analyze recurrence-free survival (RFS) [34]. Additionally in gastric cancer, HOXA transcript at the distal tip (HOTTIP) was of great importance in diagnosis and prognosis [31], while another lncRNA named lncRNAs-ZFAS1 was found in higher levels to be related to tumor metastasis [35]. Similarly, the HOX transcript antisense intergenic RNA (HOTAIR) could be used as a biomarker in breast cancer for prognosis [36].

### 3.3. Circular RNA (circRNA)

circRNAs recently emerged as a potential biomarker for diagnosis due to their stable structure, conserved consequence and extensive expression [9]. In addition, exosomal circRNAs that exist inside exosomes have a better abundance than the circRNAs in normal cells in cancer patients. For instance, in colorectal cancer, those cell lines with KRAS mutations were found to have a great decrease in the level of circRNAs in TEXs, which suggested that circRNA may be the potential diagnostic biomarker regulated by KRAS mutations [37]. In pancreatic cancer, circ-IARS was found to be involved in tumor progression, invasion and metastasis [38].

### 3.4. DNA

DNA is increasingly being used for the diagnosis and prognosis of cancer after being proved that it indeed existed in exosomes, though it is not as efficient as miRNA. Because of the short half-life and high sensitivity, stability and mutation rate, DNA has great potential in clinical applications for real-time tumor monitoring [9]. In different tumors, mutations in DNA have been regarded as biomarkers. EGFR mutations were the indicators of prognosis and diagnosis in non-small cell lung cancer (NSCLC) [39]. KRAS mutations were found both in miRNA and DNA in pancreatic cancer and colorectal cancer for diagnosis and prognosis [40,41]; specifically, the Transforming growth factor beta receptor II (TGFBR2) mutation was reported in colorectal cancer [42]. Mutations of exosomal DNA including p53, MutL homolog 1 (MLH1) and the Phosphatase and tensin homolog (PTEN) gene mutations have been reported in prostate cancer [43]. In addition, BRAF (V600E) mutations are reportedly involved in melanoma cancer [44], and the Histidyl-tRNA synthetase (HRAS) and the Epidermal growth factor receptor 2 (HER2) mutations have been found in breast cancer [45]. Until now, the exosomal DNA mutations were mainly used for prognosis and diagnosis of various type of cancers.

Nevertheless, the applications of exosome-dependent biomarkers still face many challenges. Despite the extensive use of exosomes in diagnosis and prognosis of cancer, the lack of standard methods for isolating exosome populations and the lack of heterogeneity in TEXs are still problems that exist [46]. When it comes to the exosomal nucleic acids, like DNA, difficulties in extraction and the insufficient total amount are the main hurdles, which restrain the exosomal DNA from being efficient biomarkers [47]. Though miRNA plays a central role in diagnosis, it still has a lot limitations because of the variation in miRNA levels and types; many more kinds of miRNAs have to be learned [48]. In addition, using circRNA as a biomarker also needs more research, because of the complicated and unclear mechanism of transfer, regulation and molecular selection [37]. Furthermore, mRNA is the subject of a new direction of research as a biomarker in exosomes. Though great limitations exist, recent studies have reported that exosomes have the potential to be an effective biomarker for diagnosis, especially combined with the detection of circRNA and lncRNA [49]. The contents and functions of genetic materials mentioned above in TEX are summarized in Table 1.

## 4. TEX-Mediated Abscopal Effect and Immunomodulation

### 4.1. TEX-Mediated Immunostimulatory Activities

According to the previous studies, most functions of exosomes are for immune suppression. However, recent studies have suggested that TEXs carry stimulatory molecules, in addition to the inhibitory molecules. These TEXs can deliver the stimulatory signals to distant immune cells and promote immunity, leading to eradication of established tumors. TEXs can indirectly deliver tumor antigens to dendritic cells, and then activate cytotoxic activities of CD8+ T cells and CD4+ T helper cells, resulting in suppressing tumor growth and resistance to malignant tumor development [50]. The tumor antigen and other molecules carried by TEXs include co-stimulatory molecules, MHC class II and class I molecules, tumor-associated antigens, nucleic acids, apoptotic bodies [51], intraluminal growth-promoting cytokines like epidermal growth factors (EGFR) and transforming growth factor-beta (TGF-β), heat shock proteins (HSP) 70–80 [52] and tetraspanins. For example, in melanoma, the immunostimulatory ability of exosomes is favored by the expression of MHC class I molecules and other antigens to promote an anti-melanoma response by potentiating the cytotoxic activities of CD 8+ T-cells [53]. On the other hand, heat shock proteins (HSP) are a group of common proteins that play a key role in the cell responses to environment stress. HSPs are involved in the presentation of peptide fragments to the cell surface and in generating immune responses to defend against infection and other diseases. Some preclinical studies have shown that HSP70 is the most common HSP, which is crucial in terms of stimulating immune cells and triggering anti-cancer immunity. Therefore, it has been reported that the more HSPs that exosomes carry, the greater the effect of cancer immunotherapy [54]. Besides, the TEXs with HSP-70 could activate the NK cells to facilitate an immune response. Studies have shown that they were also a new kind of shared tumor rejection antigen, which could specifically trigger MHC class I-restricted cytotoxic T cells activation [54].

All cells in the tumor microenvironment produce exosomes, and TEXs could reprogram the microenvironment to promote immune activities by directly or indirectly inducing the immunostimulatory signals [6]. Actually, many studies recently suggested that TEXs had the potential to promote differentiation and antigen-processing capabilities of dendritic cells in the tumor microenvironment, which may play a key role in dendritic cell-based cancer immunotherapies [6]. In this way, many researchers believed that TEXs were effective carriers for pulsing dendritic cells by tumor-associated antigens to improve anti-tumor response. Then the pulsed dendritic cells could induce T cells’ dependent anti-tumor response, including CD4+ and CD8+ T cells. Like in the studies of melanoma exosomes, in contrast of the most inhibitory effect on dendritic cells’ activity, a study using the B16F1 melanoma cell line showed that those exosomes may induce dendritic cell maturation, and thus stimulate the proliferation of T cells [53]. Additionally, the enrichment of HSPs on TEXs promotes the uptake of TEXs by dendritic cells, then presents it to the T cells and thereby induces an elevated immune response [54]. Dendritic cells loaded with TEXs in tumor-draining lymph nodes could promote the production of pro-inflammatory cytokines, then the level of IL-6, IFN-γ and IL-12 in the tumor microenvironment would become high, while the IL-10 was of a low level, which promoted the anti-tumor response of Th1 [6]. In addition, CD8+ T cells could be specifically activated to become tumor-specific cytotoxic lymphocytes to generate an anti-tumor response. Previous studies showed that CD8+ T cells were distinctly increased in peripheral blood and tumor tissues, and investigators found that CD8+ T cells were differentiated into cytotoxic lymphocytes when stimulated strongly by EG7 tumor cell-derived exosomes (EXOEG7)-targeted dendritic cells [55]. In addition, tetraspanins are cell-surface proteins found both on the plasma membrane and exosomes to mostly mediate cell adhesion. Therefore their major ability is forming cell-surface complexes with other cell adhesion molecules which are essential to keep immune proteins like MHC class II in an optimal conformation, so that they could play a key role in exosomal targeting to dendritic cells and the elevation of immune responses [9,54,56].

### 4.2. TEX-Mediated Abscopal Effect and Immunomodulation

**Figure 2:**
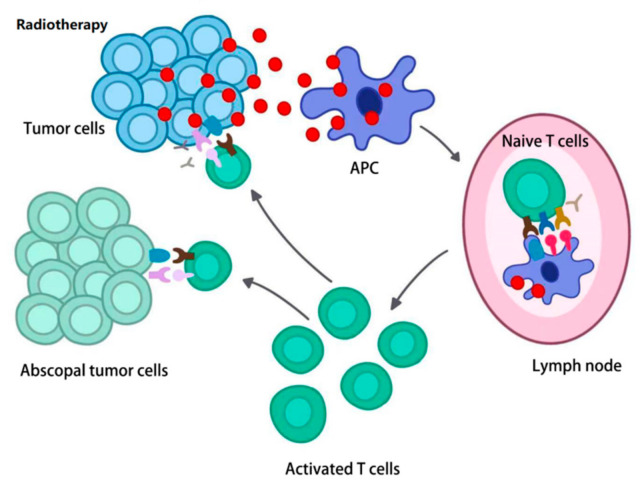
A brief mechanism of the AbE. Radiotherapy stimulates tumor cells, the TEXs are released and processed by APC, and then presented to naive T cells, which activates CD8+ T cells. Finally, activated CD8+ T cells move to the abscopal tumor cells to kill the metastasis tumor cells.

In addition to the immunotherapy, radiotherapy is another common method used for curative or palliative cancer therapy. A phenomenon called the “abscopal effect (AbE)” was first found by Golden and colleagues in clinical trials, and refers to the tumor regression that occurs in fields without irradiation distant from the local tumor area (Figure 2). Because the malignant tumors in the trials were always accompanied by metastasis to other sites, and on the basis of the expectation of powerful and sustained systemic therapeutic therapy, the AbE was further studied and enhanced for better clinical application. Studies showed that ionizing radiation in radiotherapy could induce the production of TEXs and change the exosomal secretion pattern and content of exosomes in local cells, leading to the AbE in abscopal tumor cells [57]. As the AbE was more often found in people with stronger immune systems [58], the AbE caused by radiation therapy could be explained by immune mechanisms. Radiation could induce cell death at the local area by causing DNA damage, and this process was termed “immunogenic cell death”, and then, the damage-associated molecular patterns (DAMPs), chemokines and some tumor-associated antigens were released in TEXs [4], which were processed by antigen-presenting cells including macrophages and dendritic cells to trigger the activation of CD8+ T cells; thus, the CD8+ T cells could kill the distant metastasis tumor cells through circulation [1]. Recent studies proved that the release of high-mobility group box 1 (HMGB1) and ATP were of great importance in improving the capacity of dendritic cells for antigen cross-presentation and dendritic cells maturation [59]. Additionally, HMGB1 stimulated the release of several cytokines including TNF, IL-6 and IL-8, which may be associated with the maturation and activation of antigen-presenting cells [60]. Moreover, HMGB1 could bind to toll-like receptor 2 (TLR2) and TLR4 to promote antigen presentation and elicit an inflammatory effect [61,62]. Released DNA from damaged tumor cells could induce the secretion of type I IFN-γ through a stimulator of interferon genes in the cyclic guanosine monophosphate-adenosine monophosphate synthase signaling pathway [63]. In addition, type II IFN-γ was also released to upregulate the MHC class I and vascular cell adhesion molecule 1 (VCAM1) expression to enhance the tumor antigen presentation [64]. Released chemokines like CXC-motif chemokine ligand 9 (CXCL9), CXCL10 and CXCL16 were shown to upregulate the adhesion molecules expression, such as intercellular adhesion molecule 1 (ICAM1) and E-selectin for promoting arrested by T cells [4,65].

On the other hand, radiotherapy combined with immunotherapy and the mechanism of the AbE had the potential of enhancing the therapeutic effect. For example, in a study by Poggi et al., a TEX-related mouse model named TRAMP-C2 (mouse tumor cell line) was proposed. The TRAMP-C2 cells lacking exosomal PD-L1 were not only unable to grow, but the contralateral tumor growth was significantly reduced when the cells were injected into the other side of the mouse, suggesting that the lack of PD-L1 in tumor cells could induce the AbE. This study indicated that PD-L1 on TEXs can be used as a target for anti-tumor therapy [66]. Studies in recent years have been mostly focused on the immune checkpoint inhibitors, including cytotoxic T lymphocyte-associated antigen 4 (CTLA-4) blockades and programmed cell death-1 (PD-1)/PD-L1 blockades, as the PD-L1 and CTLA-4 are all tumor-associated antigens on TEX, and CTLA-4 and PD-1 were both molecules that could inhibit the immune response [65]; thus, many studies have shown the anti-CTLA-4 [67], anti-PD-1/PD-L1 and anti-CD-137 (also known as 4-1BB or tumor necrosis factor receptor superfamily member 9 (TNFRSF9) that has been induced by lymphocyte activation (ILA)) antibodies) [68] all act as immune checkpoint inhibitors. Unfortunately, they were not therapeutically effective when they were used alone. However, and encouragingly, a combination treatment with the immune checkpoint inhibitors and immunostimulatory molecules, such as recently reported exosomes, remarkably enhanced the AbE and anti-tumor efficacy of radiotherapy. Moreover, the radiotherapy-induced AbE could be enhanced by exosomes derived from activated mesenchymal stem cells (MSCs) [69]. MSCs extensively exist in many tissues including cancer tissues, whose main function is to be responsible for wound healing. This function is dependent on the exosomes derived from MSCs. Research has proved that loading with anti-apoptotic miRNAs and growth factor receptor mRNAs favors wound healing and angiogenesis [70]. ANXA1 was a kind of annexin in exosomes released by activated MSCs, which was regarded as an anti-inflammatory molecule, while the tumor-induced inflammatory molecules would promote the tumor growth and metastasis [71]. By inducing apoptosis of neutrophils and removing dead neutrophils [72], microenvironment homeostasis and anti-inflammatory effects could be achieved. Besides, several recent animal studies suggested that the combination of radiotherapy and immune checkpoint inhibitors like anti-PD-L1 was more efficient [73]. Radiotherapy activates the AbE first: when PD-1 on activated CD8+ T cells binds to the PD-L1 from TEX carried by APC, it inactivates CD8+ T cells utilizing anti-PD1 antibodies, which facilitates the maintenance of CD8+ T cell activation [74]. In addition, although some studies have supported this view, others have suggested that the therapeutic effect of the combination of these two therapies is related to the dose of radiation [75,76] and different types of radiotherapy, like stereotactic body radiotherapy (SBRT) [73], and for different tumors, the dose of radiotherapy that could achieve the best therapeutic effect was different [76]. However, there are some different results in other studies and clinical trials. In the newest clinical trials by Kazandijian et al. [77] and the study by Mcbride et al. [78], the combined treatment did not work as expected. Additionally, some of the side effects of radiation therapy are also worrisome, for instance, standard or high radiation doses can lead to the exposure or release of tumor-associated antigens [79]. Moreover, radiotherapy can increase tumor PD-L1 expression, MHC I expression, exhaustion of CD8+ T cells and changes in the tumor microenvironment, which may enhance the resistance to anti-CTLA-4 and anti-PD-L1/PD-1 [80]. In summary, the therapeutic effect of radiotherapy may be enhanced by the combination with immunotherapy, and though it is possible to observe an AbE induced by combined therapy during treatment, this was extremely rare. Further research is needed to explore the exact mechanism of the AbE induced by radiotherapy, and the different doses and radiotherapy methods for each kind of tumor need to be confirmed. At the same time, it is also necessary to explore the immune mechanism of immune checkpoint inhibitors and the clinical feasibility of their combination with radiotherapy, so as to provide guidance for the future development of more cancer treatment, where the AbE may play a key role in clinical anti-cancer therapy.

## 5. Tumor-Derived Exosomes as Biomarkers

By participating the promotion of angiogenesis, stromal rebuilding, activating the signaling pathway through growth factor/receptor transfer, drug resistance and intercellular genetic exchange, TEXs could be regarded as the central mediator in the tumor microenvironment [81].

Recent studies indicated that being biomarkers was the most important biological function of TEXs [56]. A biomarker is a kind of indicator of physiological and biological states, which in cancer is used for diagnosis, prognosis, indicating tumor progression and therapeutic response [82]. The exosomes and their content could stably exist in biological fluids like serum, urine and saliva; thus, they could serve as “liquid biopsy” biomarkers of different types of cancers, which is a newly developed non-invasive approach [83]. The molecules contained in TEXs represent the content of parent cells, which may relate to the condition of the whole tumor, intercellular communication and the tumor microenvironment. What makes TEXs appropriate biomarkers is the protection of TEXs’ membranes, which help prevent the content in TEXs, especially miRNAs, from being degraded [84]. These TEXs should have a higher specificity and sensitivity [56]. The molecules they transfer mainly include nucleic acids, glycoconjugates, proteins and lipids, and all of them could work as biomarkers. Because of their expression levels being significantly higher than normal cells, exosomal miRNA and exosomal proteins have been more frequently used as promising diagnosis biomarkers for lung cancer, hepatocellular carcinoma, pancreatic cancer and gastrointestinal cancer (as summarized in Table 2) [9]. miRNA is a kind of molecule with the ability of regulating post-transcription and may be involved in immune suppression, because of the capacity of blocking the whole signaling pathway by binding to suppressor complex. Previous studies also proved that miRNAs were of great importance in cancer detection, histotypes discrimination and prognosis [85]. Studies have shown that contents in TEXs, such as variable proteins and nucleic acids including miRNA, lncRNA, circRNA and DNA, could be detected as biomarkers for diagnosis and prognosis by the methods of transcriptomics and proteomics [16]. In non-small cell lung cancer (NSCLC) studies, some of the proteins like NY-ESO-1 and placental alkaline phosphatase (PLAP) had a strong association with survival [86]. Other studies using multivariate extracellular vesicle array found that CD91, CD317 (also referred as Tetherin or BST2, or HM1.24 antigen) and EGFR were specific antigens of NSCLC as biomarkers for diagnosis [87]. Additionally, through the analysis of proteomic mass spectrometry, there was a higher level of leucine-rich alpha-2-glycoprotein 1 (LRG1) expression in urinary exosomes detected in cancer patients [88]. CD171, CD151 and tetra-spanin 8 were regarded as potential biomarkers for the diagnosis of NSCLC [86]. The extracellular matrix protein 1 (ECM1) and alpha-2-HS-glycoprotein were elevated in serum exosomes, and with the combination of carcinoembryonic antigens, they could be more accurate for diagnosis [89].

In pancreatic cancer patients, glypican-1 (GPC-1), a membrane-anchored proteoglycan, and GPC-1+ exosomes were found with a higher level in serum than in healthy people, and they had great sensitivity and specificity in the diagnosis of early pancreatic cancer [90]. In addition, GPC-1+ exosomes were observed to be strongly associated with the survival of patients with or without operation [9]. The migration inhibitory factor was another biomarker which had a higher level of expression in stage I patients with migration potential [91]. Vimentin itself can be used in the diagnosis of pancreatic cancer and was found to decrease after being given the treatment of gemcitabine, which could be used as indicator of prognosis [92].

In prostate cancer, PCA3 and TMPRSS2:ERG are two proteins used as biomarkers for diagnosis, which exist in urinary exosomes [93]. EGFR also exists in urinary exosomes, which may be a new kind of indicator of tumor progression [94]. In gastric cancer, similar to prostate cancer, EGFR was of a higher level in exosomes [95], and recently, tripartite motif-containing 3 (TRIM3) was found to be decreased in the serum of gastric cancer patients, making exosomal TRIM3 a potential biomarker of gastric cancer [96].

In colorectal cancer, recent studies found that proteins in serum-purified exosomes (SPE) were important biomarkers for tumor metastasis. For instance, hepatocyte growth factor-regulated tyrosine kinase substrate (HGS) was a biomarker for poor prognosis [97]. Besides, CD147 carried by TEX plays a key role in tumor progression, tumor invasion and metastasis; it is the biomarker of diagnosis and prognosis for colorectal cancer [98], and the expression of CD147 is usually associated with decreased survival [99].

Though exosomal proteomics methods are of great importance in tumor diagnosis and prognosis, there are still difficulties in the quality requirements of exosomes because of the low level of exosomal-specific proteins in serum [90]. Moreover, only a small fraction of the total protein has been found to be useful for being a biomarker, whereas the specific functions of other proteins inside the exosomes still remain unknown.

**Table 2:** Biomarkers.

Biomarkers	Sources	Tumor Type	Significance	Reference
NY-ESO-1, PLAP	EV microarray	NSCLC	Strong association with survival	[86,87]
CD91, CD317	Multivariate extracellular vesicle array	NSCLC	Diagnosis	[87]
EGFR	EV microarray, Western blotting and ELISA	NSCLC, prostate cancer, gastric cancer	Diagnosis and potential indicator of tumor progression	[87,94,95]
LRG1	Proteomic mass spectrometry	NSCLC	Diagnosis	[88]
CD171, CD151, tetra-spanin 8	EV microarray	NSCLC	Potential biomarkers for diagnosis	[86,87]
ECM1, alpha-2-HS-glycoprotein	Western blotting	NSCLC	Diagnosis	[89]
GPC-1	Ultracentrifugation	Pancreatic cancer	Early diagnosis	[90]
Migration inhibitory factor	Ultracentrifugation	Pancreatic cancer	Migration potential monitoring and prognosis	[91]
Vimentin	Ultracentrifugation	Pancreatic cancer	Diagnosis and prognosis	[92]
PCA3, TMPRSS2: ERG	PCR analysis	Prostate cancer	Diagnosis	[93]
TRIM3	RT-PCR	Gastric cancer	Potential diagnosis biomarker	[96]
HGS	Proteomics analysis	Colorectal cancer	Poor prognosis	[97]
CD147	Western blotting, RT-PCR	Colorectal cancer	Diagnosis and poor prognosis	[98,99]

## 6. Role of Tumor-Derived Exosomes in Cancer Therapy

### 6.1. TEX-Targeted Cancer Therapy

Since TEXs play a role in tumor progression, drug resistance and metastasis, a therapeutic method was developed to block the circulation of TEXs in blood for the treatment of cancer patients. This method is called adaptive dialysis-like affinity platform technology (ADAPT™). When the blood from a cancer patient is transferred through this system, the plasma specimen factor interacts with immobilized affinity agents through porous fibers to target molecules that can be selectively absorbed, while other blood cells and contents can go through the system [100]. Though it is a promising method, it still needs to be optimized to achieve therapeutic efficacy in clinical application, since TEXs are abundant in cancer patients [100]. In addition, another method was found by Gamperl in 2016, which was a low-molecular weight heparin called Tinzaparin that can induce the release of the tissue factor pathway inhibitor (TFPI) from tumor cells, and the recombinant TFPI then inhibits the TEXs inducing tumor cell migration [101].

### 6.2. TEX-Based Drug Delivery for Cancer Chemotherapy

TEXs can be used for targeting specific tissues and drug delivery. For example, Doxorubicin carried by TEXs can be delivered to the target cancer tissue, like that of colorectal or breast cancers, which has shown a higher efficacy in repressing tumor progression and metastasis [102]. Because of the small size, TEXs can escape from the phagocytosis by the mononuclear phagocyte system, making TEXs easy to extravasate from the blood vessel and easy to diffuse in tumor tissues. Additionally, the membrane of TEXs is made of phospholipid bilayers, which can directly diffuse with the targeting cells, resulting in cellular internalization of the encapsulated drug carried by TEXs. Furthermore, TEXs had the least cellular toxicity in cancer therapy since they were derived from autologous tumor cells [16]. TEXs also demonstrated the ability to target specific tissues and organs because of their specific cell tropism [13], which was also the base of delivering drugs to the cancer tissue. The modification of the exosomes with targeting ligands can reprogram the TEXs for targeting cancer tissues in more specific manor. Taken together, TEXs have demonstrated their great clinical potential in being drug carriers for cancer therapy. However, the problems in clearance and distribution still need to be considered, and their clinical use also needs further study [16].

### 6.3. TEX-Based Cancer Vaccine and Immunotherapy

**Figure 3:**
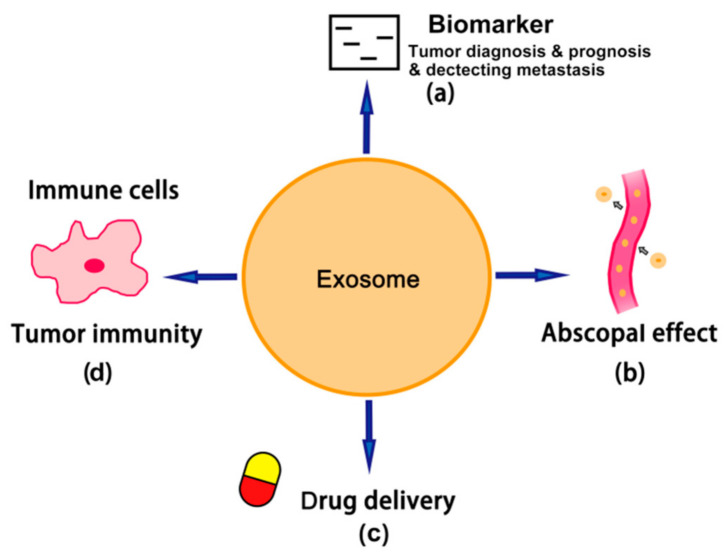
The role of exosomes in cancer. (**a**) Exosomes can be used as biomarkers. Genetic materials like miRNA, circRNA, lncRNA and DNA inside the exosomes can be used as useful tools for tumor diagnosis, prognosis and detecting metastasis. (**b**) Exosomes actively participate in the abscopal effect and improve the anti-tumor therapeutic effect. (**c**) Exosomes can be used for drug delivery in chemotherapy. (**d**) Exosome-pulsed dendritic cells can activate an immune response and are emerging anti-tumor vaccines.

As shown in Figure 3, dendritic cells pulsed by TEXs are a promising new generation of anti-tumor vaccines [103]. TEXs could stimulate dendritic cells and then activate cytotoxic lymphocytes. Multiple clinical trials have already proved that these type of new cancer vaccines have greater efficacy and safety than conventionally used tumor lysates or normal exosome-pulsed dendritic cells [104]. According to the clinical therapeutic study, treatment with the TEX-loaded dendritic cells displayed a higher efficacy in suppressing tumor growth and upregulating anti-tumor immunity, implying that dendritic cells loaded with TEXs are promising therapeutics with the least adverse effects in clinical application [105]. In the future, TEXs may become the potential ideal adjuvant component by reprogramming the ligands and antigens on them. Research conducted in 2017 showed that, in leukemia, a stronger anti-tumor response could be induced by pulsed dendritic cells through silencing the TGF-1 expression by operated TEXs [106]. TEXs have the ability to shift the immunosuppressive microenvironment towards an immunostimulatory environment when TEXs are reprogrammed with co-stimulatory ligands/receptors and phosphatidyl serine, which ensure the strong stimulating signals and effective uptake of antigen by dendritic cells [6].

## 7. Conclusions

### TEXs—A Double-Edged Sword

In summary, the TEXs originated from tumor cells possess the ability to suppress anti-tumor immune responses and promote tumor progression and metastasis. TEXs suppress anti-tumor immune responses because of the inhibition of immune effector cells and the interference of cancer therapy, directly or indirectly induced by immunosuppressive ligands carried by TEXs [6]. In addition, TEXs carrying immunosuppressive ligands also can inhibit the anti-tumor function of NK-92 cells in leukemia adoptive cell therapy, and then restrict the therapeutic effect [107]. Moreover, TEXs can activate immune suppressor Tregs cells [108]. Besides, some therapeutic antibodies used in cancer therapies could also be affected by the tumor-associated antigens carried by TEXs, restricting the antibodies to diminish their effect, even blocking their therapeutic pathway [109].

On the other hand, TEXs may also contribute to the AbE, an emerging mechanism of anti-tumor immunity (Figure 2). TEX-mediated AbE may also synergize the therapeutic effects of immunotherapy and radiotherapy for cancer patients. Moreover, TEX-pulsed dendritic cells may become the new generation of cancer vaccines. However, the optimal radiation dose to stimulate the best TEX-based AbE, as well as the optimization of the combination of TEX/AbE-mediated immunotherapy, the control of the TEX-mediated drug delivery system and the development of TEX-based biomarkers are all challenges we are facing, and are the urgent topics that still need to be further investigated [65].

## Figures and Tables

**Table 1:** TEX-containing genetic material associated with cancer progression.

Genetic Material	Contents and Functions	Reference
miRNA	miR-103: Biomarkers for metastasis of Hepatocellular carcinoma. miR-638: Recurrence of Hepatocellular carcinoma. miR-1229, miR-1246, miR-150, miR-223, miR-23a: Diagnosis of colorectal cancer. miR-17-92a, miR-19a: Recurrence of colorectal cancer. miR-217, miR-4257: Recurrence of lung cancer. miR-51, miR-373: Poor diagnosis of lung cancer. miR-208a, miR-1246: Biomarkers of therapeutic effect of lung cancer. miR-4644, miR-1246: Poor diagnosis of pancreatobiliary tract cancer. KRAS mutation: Diagnosis of pancreatic cancer and colorectal cancer.	[22,23,24,25,26,27,28,29]
lncRNA	lnc-sox2ot, lnc-h19, lncRNA-ARSR: Prognosis and survival rate of hepatocellular carcinoma. lncRNA-UCA1: Diagnosis and tumor progression monitoring of bladder cancer. HOTTIP: Diagnosis and prognosis of gastric cancer. lncRNAs-ZFAS1: Biomarkers for metastasis of gastric cancer. HOTAIR: Prognosis of breast cancer.	[22,30,31,32,33,35,36]
circRNA	circ-IARS: Biomarkers for tumor progression, invasion and metastasis of pancreatic cancer.	[9,37,38]
DNA	EGFR mutation: Prognosis and diagnosis of NSCLC. KRAS mutation: Diagnosis and prognosis of pancreatic cancer; regulation of circRNA as diagnostic biomarker in colorectal cancer. TGFBR2 mutation: Diagnosis of colorectal cancer. P53 mutation, MLH1 mutation, PTEN mutation: Diagnosis of prostate cancer. BRAF mutation: Diagnosis of melanoma cancer. HRAS mutation, HER2 mutation: Diagnosis of breast cancer.	[9,36,37,39,40,41,42,43,44,45,46,47,48]

## Data Availability

Not applicable.
